# Laparoscopic partial pericystectomy for an unusual subdiaphragmatic hydatid cyst adjacent to the adrenal gland in a 13-year-old patient: a case report

**DOI:** 10.1097/RC9.0000000000001161

**Published:** 2026-10-21

**Authors:** Sevinj Salahova, Anar Namazov, Rasim Bayramov, Aghamaliyeva Aytan Jafar, Nuru Bayramov

**Affiliations:** a Department of Surgical Diseases I, Azerbaijan Medical University, Teaching Surgical Clinic, Baku, Azerbaijan; b Department of Radiology, Azerbaijan Medical University, Teaching Surgical Clinic, Baku, Azerbaijan

**Keywords:** case report, hydatid cyst, laparoscopic pericystectomy, subdiaphragmatic location

## Abstract

**Introduction:**

Hydatid disease rarely involves the adrenal region, particularly in pediatric patients, making diagnosis and surgical management challenging due to the complex anatomical relationships in the subdiaphragmatic space. This case highlights the feasibility of laparoscopic management of a hydatid cyst in an unusual subdiaphragmatic location, adjacent to the adrenal gland, in a child.

**Presentation of the case:**

A 13-year-old boy from an endemic region presented with intermittent epigastric pain without systemic symptoms. Ultrasonography demonstrated a cystic lesion beneath the left hemidiaphragm, and contrast-enhanced computed tomography revealed a 60–63 mm thick-walled cyst between the stomach and spleen, closely adherent to the left crus of the diaphragm and the left adrenal gland. The patient underwent laparoscopic partial pericystectomy with preservation of the adrenal gland. Histopathological examination confirmed hydatid disease. The postoperative course was uneventful, and no clinical or ultrasonographic evidence of recurrence was detected during the 3-month follow-up period.

**Clinical discussion:**

Hydatid cysts adjacent to the adrenal gland are rare in pediatric patients and present diagnostic and surgical challenges due to their complex anatomical location. This case demonstrates the technical feasibility of laparoscopic adrenal-sparing partial pericystectomy in a carefully selected pediatric patient, when meticulous surgical technique is applied.

**Conclusion:**

In selected pediatric patients with hydatid cysts in unusual subdiaphragmatic locations, laparoscopic partial pericystectomy may provide a feasible organ-preserving treatment option. Longer follow-up is required to assess long-term outcomes and recurrence.

## Introduction

Hydatid disease is a zoonotic parasitic infection caused by *Echinococcus granulosus* and remains a significant health problem in endemic regions, including the Middle East, Eastern Europe, and parts of Central Asia^[^1,2^]^. Humans act as accidental intermediate hosts after ingestion of parasite eggs, most commonly through contaminated food or close contact with infected animals^[^1^]^.

HIGHLIGHTS

- A hydatid cyst adjacent to the adrenal gland is an exceptionally rare pediatric presentation.
- Computed tomography was essential for accurate preoperative assessment and surgical planning.
- Laparoscopic adrenal-sparing partial pericystectomy was performed safely.
- The use of 0.05% chlorhexidine digluconate minimized the risk of intraoperative cyst dissemination.
- The patient recovered uneventfully without recurrence during follow-up.

The liver is the most frequently affected organ, accounting for approximately 60–70% of cases, followed by the lungs^[^2,3^]^. Hepatic hydatid cysts may remain asymptomatic for a long period. Depending on their size and location, they may present with abdominal pain, mass effect, or biliary complications^[^3^]^.

Despite advances in ultrasonography and computed tomography (CT), diagnosis of hydatid disease may remain challenging in atypical locations, particularly when the lesion is closely related to the adrenal gland. In such cases, radiological findings may overlap with those of other cystic lesions, and definitive diagnosis is often confirmed by histopathological examination. Surgical treatment remains the cornerstone of management, with the choice of procedure depending on cyst location, anatomical relationships, and the feasibility of safe removal while minimizing the risk of spillage and recurrence^[^4–6^]^. Hydatid cysts adjacent to the adrenal gland are exceptionally uncommon, especially in pediatric patients, and only a limited number of cases have been reported in the literature^[^7–11^]^. Their unusual anatomical location poses important diagnostic and technical challenges because of the proximity to the diaphragm, stomach, spleen, and adrenal gland. This case highlights the diagnostic value of preoperative imaging and the feasibility of laparoscopic adrenal-sparing partial pericystectomy in a child with a hydatid cyst in this unusual location.

This case has been reported in accordance with the SCARE 2025 criteria.

## Case presentation

A 13-year-old boy weighing 53 kg presented with intermittent abdominal pain localized to the epigastric region. The symptoms were not accompanied by fever, vomiting, or weight loss. The patient lived in an endemic region for echinococcosis. The past medical history was unremarkable. On physical examination, the patient was in good general condition. Mild epigastric tenderness was noted without signs of peritoneal irritation.

Abdominal ultrasonography demonstrated a well-defined anechoic cystic lesion measuring approximately 45 mm beneath the left hemidiaphragm. Contrast-enhanced CT revealed a 60–63 mm, thick-walled cyst located between the stomach and spleen, closely adherent to the left crus of the diaphragm and the left adrenal gland, without evidence of invasion into adjacent organs. The cyst was located in close proximity to the left adrenal gland; however, its exact relationship to the adrenal gland and its organ of origin could not be conclusively established on the available preoperative CT images. The lesion exhibited imaging characteristics suggestive of hydatid disease, including a well-defined capsule and internal cystic architecture.

Figure 1 illustrates the preoperative CT findings and the anatomical relationship of the cyst to the surrounding structures. Laboratory investigations showed mildly elevated serum bilirubin levels, while other hematological and biochemical parameters were within normal limits.

**Figure 1.**
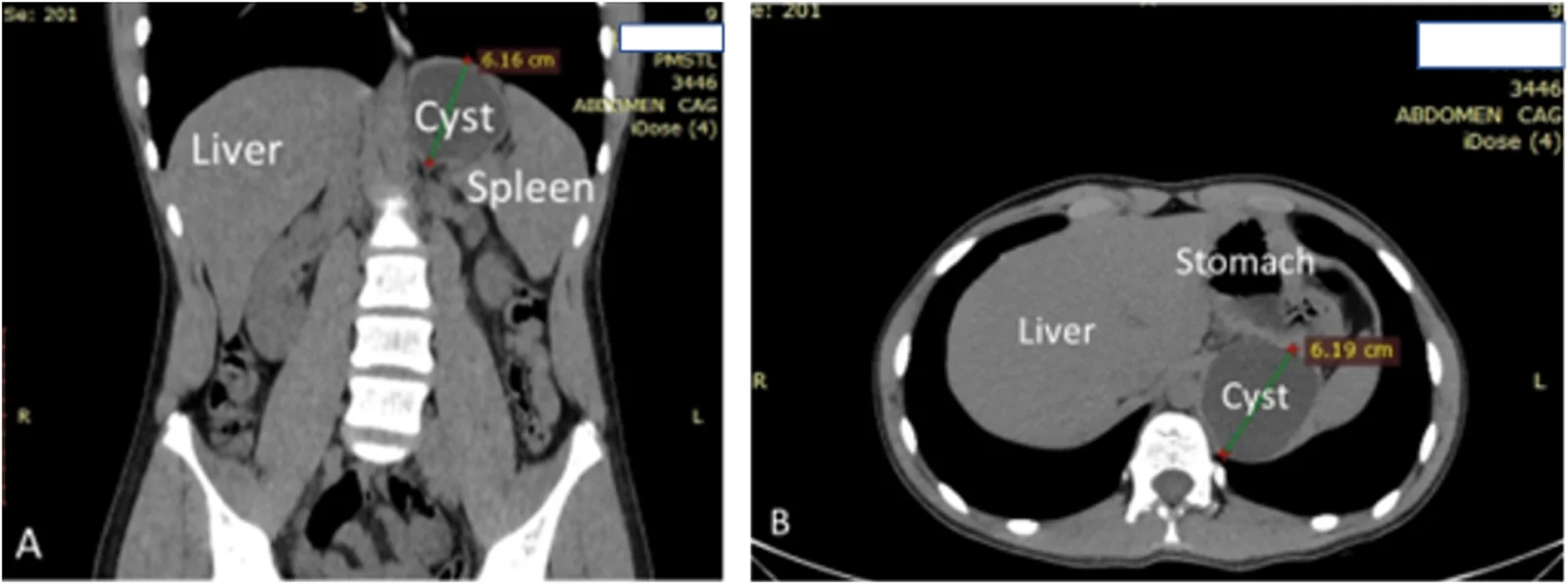
(A) Contrast-enhanced computed tomography demonstrating a well-defined, thick-walled cystic lesion measuring approximately 60–63 mm in the left subdiaphragmatic region (arrow). The cyst is located between the stomach and spleen and is closely adherent to the left crus of the diaphragm and the left adrenal gland. (B) Coronal CT reconstruction illustrating the anatomical relationship of the hydatid cyst to the adjacent organs, facilitating preoperative surgical planning. CT, computed tomography.

The preoperative differential diagnosis included an adrenal cyst, pancreatic pseudocyst, splenic epithelial cyst, retroperitoneal cystic lesion, and other benign cystic masses arising in the left subdiaphragmatic region. However, residence in an endemic area, together with the imaging findings, made hydatid disease the most likely diagnosis and supported surgical treatment.

Considering the lesion’s size, its proximity to the diaphragm and adrenal gland, and the risk of rupture or secondary infection, surgical treatment was indicated.

### Surgical procedure

On 8 December 2025, the patient underwent laparoscopic partial pericystectomy. A 10 mm supraumbilical port was placed for the laparoscope, along with two 5 mm working ports. Dense adhesions involving the stomach, spleen, left crus of the diaphragm, and left adrenal gland were carefully dissected. Following careful adhesiolysis, the left adrenal gland was identified intraoperatively as a separate anatomical structure adjacent to the cyst. Direct visualization confirmed that the cyst did not originate from the adrenal gland, which was preserved intact during the procedure.

After adhesiolysis, the operative field was carefully isolated with gauze pads to minimize contamination and to protect the surrounding tissues from exposure to cyst contents and the scolicidal solution. The cyst was punctured, and its contents were aspirated before opening the cyst cavity. After evacuation of the hydatid material, the residual cavity was irrigated with 0.05% chlorhexidine digluconate solution as a scolicidal agent. The exact duration of exposure was not documented in the operative record and, therefore, could not be reliably determined retrospectively. Chlorhexidine gluconate has previously demonstrated protoscolicidal activity at low concentrations during hydatid cyst surgery^[^12^]^. Partial pericystectomy was then performed, with preservation of the left adrenal gland and surrounding structures. Throughout the procedure, particular attention was paid to avoiding intraoperative rupture and spillage of cyst contents. The operative field was irrigated, hemostasis was achieved, and the resected cyst wall was submitted for histopathological examination.

Figures 2 and 3 demonstrate the key intraoperative findings. Because of the close anatomical relationship between the cyst, diaphragm, spleen, and left adrenal gland, careful laparoscopic dissection was essential to preserve adjacent organs and achieve complete evacuation without injury.

**Figure 2.**
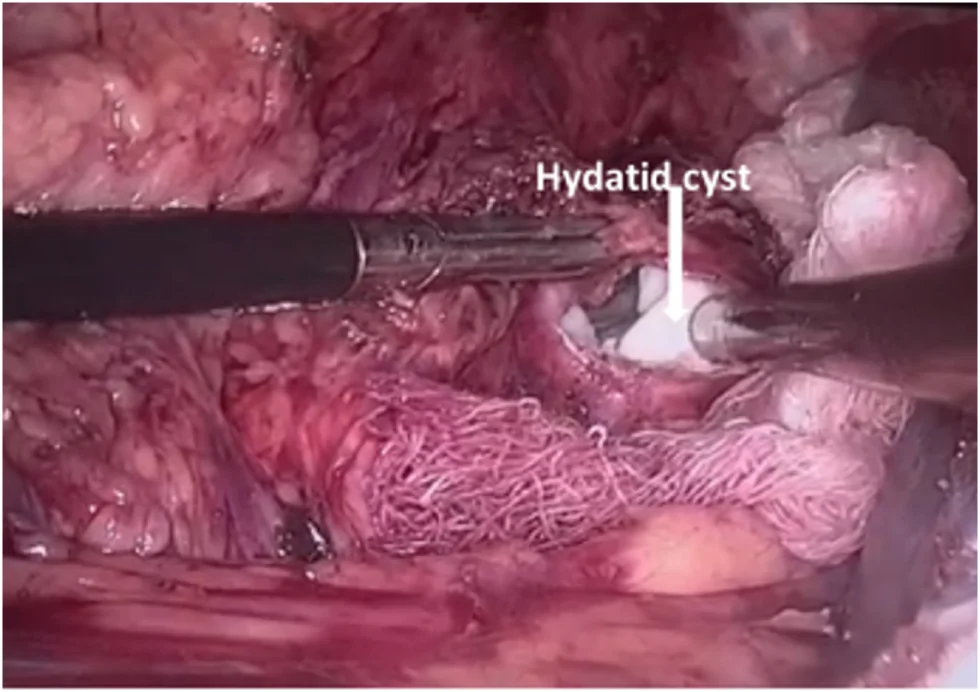
Intraoperative laparoscopic view showing the hydatid cyst after controlled opening. The white arrow indicates the hydatid cyst. The germinal membrane and hydatid contents are visible following careful evacuation, with meticulous protection of the operative field to minimize the risk of cyst spillage.

**Figure 3.**
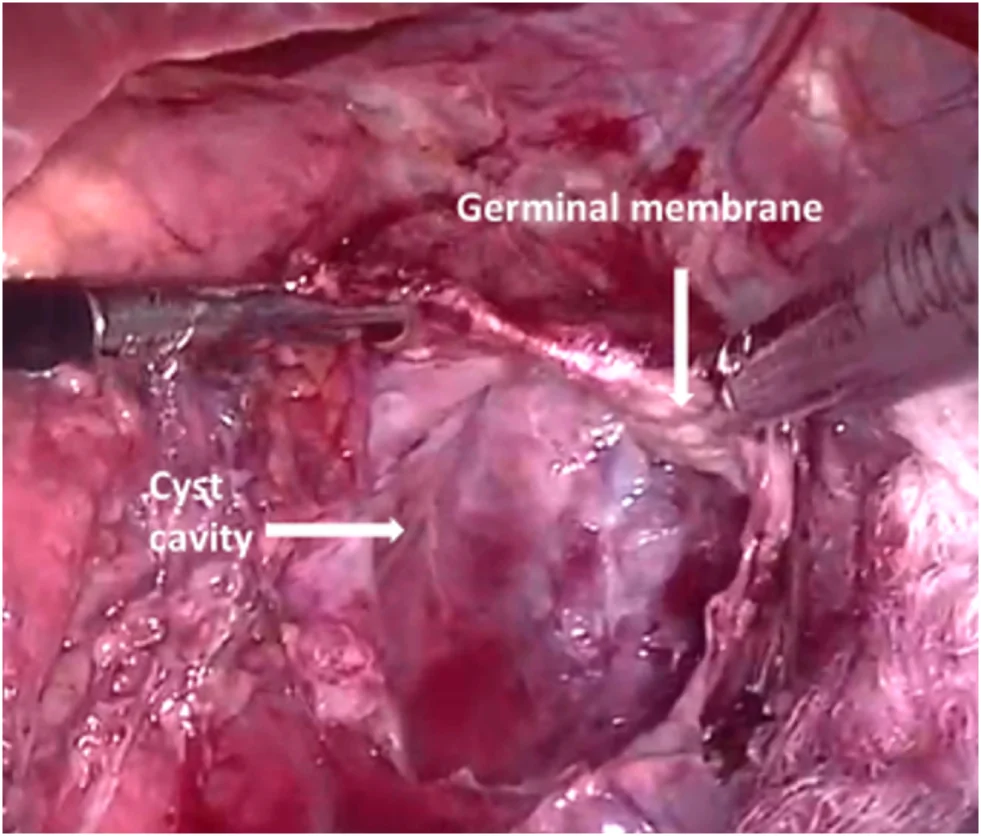
Intraoperative laparoscopic view of the hydatid cyst, showing the germinal membrane (indicated by the white marker) during laparoscopic partial pericystectomy.

### Histopathological findings

Histological examination of the excised cyst wall confirmed the diagnosis of hydatid disease. The specimen showed a laminated membrane with fragments of the germinative layer. Multiple structures consistent with daughter cysts were identified. The surrounding fibrous pericyst showed chronic inflammatory changes. These findings confirmed the diagnosis of cystic echinococcosis.

### Postoperative course

The patient remained clinically stable postoperatively. Because a residual cavity remained after partial pericystectomy, the drain was kept in place for postoperative monitoring. The patient was discharged on postoperative day 1 with the drain *in situ*. The patient and his caregivers were provided with detailed instructions regarding drain care, monitoring of the amount and character of the drainage, and warning signs requiring medical reassessment. The drain was removed approximately 2 weeks after surgery, when the drainage had decreased to a minimal amount, and there were no clinical signs of infection or other postoperative complications. According to information obtained from the patient’s caregivers, albendazole had been administered before the patient was referred to our surgical unit for operative treatment. The exact dose and duration of the preoperative albendazole therapy could not be reliably verified from the available medical records. Postoperatively, albendazole was administered at a dose of 200 mg twice daily for 1 month, followed by a 2-week treatment-free interval and a second 1-month cycle. Follow-up with ultrasonography and routine laboratory investigations at 2 weeks, 1 month, and 3 months showed no clinical or ultrasonographic evidence of recurrence and no evidence of albendazole-related hepatotoxicity. No clinical signs suggestive of adrenal dysfunction were observed during the 3-month follow-up period. However, this limited follow-up duration is insufficient to assess long-term recurrence.

## Discussion

Hydatid disease predominantly affects the liver and lungs, whereas adrenal involvement accounts for less than 1% of cases of cystic echinococcosis^[^7,8^]^. Hydatid cysts adjacent to the adrenal gland are exceptionally uncommon in pediatric patients, with only isolated cases reported in the literature^[^8–11^]^. Because of their unusual anatomical location, these lesions may mimic cystic lesions of the adrenal gland, pancreas, spleen, or retroperitoneum, creating diagnostic and therapeutic challenges^[^8,10,13^]^. Therefore, hydatid disease should always be included in the differential diagnosis of cystic masses in children from endemic regions.

Recent reports continue to demonstrate the rarity and heterogeneous presentation of hydatid cysts involving the adrenal, periadrenal, and retroperitoneal regions. Only a limited number of pediatric cases have been reported, whereas most published cases involve young or middle-aged adults. The anatomical location, surgical approach, and outcomes of selected recent cases reported over the last 5 years are summarized in Table 1^[^8,11,14–16^]^.

**Table 1:** Selected recently reported cases of hydatid cysts in adrenal, periadrenal, and retroperitoneal locations.

Reference	Age/sex	Location	Cyst size	Surgical approach	Outcome
Tajmalzai & Aien, 2025^[^8^]^	18/M	Right adrenal gland	6.2 × 5.1 × 4.5 cm	Surgical excision	Favorable outcome
Feizi *et al*, 2025^[^11^]^	22/M	Right adrenal region	–	Complete surgical excision	Favorable postoperative outcome
Haymoun *et al*, 2025^[^14^]^	13/F	Left retroperitoneal	20 × 10 × 8 cm	Endocystectomy	No recurrence during follow-up
Yadav *et al*, 2024^[^15^]^	39/M	Left suprarenal retroperitoneal region	16.2 × 10.5 × 13.5 cm	Open partial cystectomy	Favorable postoperative outcome
Pediatric case, 2026^[^16^]^	8/F	Isolated retroperitoneal cyst with renal involvement	12 × 10 cm	Nephron-sparing cystectomy	No recurrence at 12 months

Compared with recent reports, the present case is distinguished by the young age of the patient and the unusual left subdiaphragmatic location of the cyst, closely adjacent to but not originating from the adrenal gland. Furthermore, successful laparoscopic partial pericystectomy allowed preservation of the adrenal gland and surrounding structures. These features highlight the importance of careful preoperative anatomical assessment and support a minimally invasive, organ-preserving approach in carefully selected pediatric patients.

Diagnosis relies primarily on imaging studies. Ultrasonography is usually the initial imaging modality, whereas contrast-enhanced CT is essential for surgical planning^[^4,13^]^. In our patient, CT demonstrated the location of the cyst and its close relationship to adjacent organs and facilitated surgical planning; however, the precise relationship to the left adrenal gland and the organ of origin could not be conclusively established preoperatively. Histopathological examination confirmed the diagnosis^[^8–11^]^.

Surgery remains the treatment of choice for symptomatic hydatid cysts and lesions with an increased risk of rupture or compression of adjacent organs^[^5,6^]^. Although open surgery has traditionally been the standard approach for technically demanding hydatid cysts, laparoscopic surgery is a safe and effective alternative in selected patients^[^8,9,17^]^. The laparoscopic approach offers shorter recovery and better cosmetic outcomes, particularly in pediatric patients^[^8,17^]^. The choice of surgical technique should be individualized according to the anatomical location of the cyst and its relationship with adjacent organs. Total cystectomy may not be feasible because dense adhesions increase the risk of intraoperative injury^[^5,6^]^. PAIR (puncture, aspiration, injection, and reaspiration) is reserved for selected, uncomplicated hepatic cysts and is not routinely recommended for lesions with complex anatomical relationships, such as those observed in the present case^[^5,6^]^. Considering the firm adhesions between the cyst, diaphragm, spleen, and left adrenal gland, laparoscopic adrenal-sparing partial pericystectomy was selected as an organ-preserving surgical approach in this patient.

One of the major concerns during laparoscopic treatment of hydatid disease is intraoperative cyst rupture with dissemination of viable protoscolices, which may result in recurrence or secondary echinococcosis^[^5,6^]^. To minimize this risk, careful operative technique is essential. In the present case, the operative field was isolated, the cyst contents were evacuated, and the residual cavity was irrigated with 0.05% chlorhexidine digluconate solution before laparoscopic partial pericystectomy. Particular attention was paid to preventing spillage while preserving the left adrenal gland and surrounding structures. No intraoperative complications occurred. The postoperative course was uneventful, and albendazole therapy was administered as described above. No clinical or ultrasonographic evidence of recurrence was detected during the available 3-month follow-up period. However, the short duration of follow-up precludes drawing conclusions regarding long-term recurrence or treatment effectiveness.

This case has several limitations. First, the available follow-up period was limited to 3 months, which is insufficient to assess long-term recurrence or treatment effectiveness. Second, although the diagnosis of cystic echinococcosis was confirmed histopathologically, a histopathological image was not available for inclusion in the report. Third, the complete original CT dataset was unavailable, limiting retrospective radiological reassessment of the precise anatomical relationship between the cyst and the left adrenal gland; therefore, the non-adrenal origin of the cyst was established primarily by direct intraoperative assessment. Finally, although the patient’s body weight at admission was confirmed to be 53 kg, the exact dose and duration of albendazole therapy administered before referral could not be reliably verified from the available medical records.

## Conclusion

Hydatid cysts adjacent to the adrenal gland should be considered in the differential diagnosis of subdiaphragmatic cystic lesions in children from endemic regions. Preoperative imaging and careful laparoscopic technique may facilitate adrenal preservation while minimizing the risk of cyst spillage. In selected patients, laparoscopic partial pericystectomy may provide a feasible organ-preserving approach when preservation of adjacent structures is technically achievable. However, longer-term follow-up is required to assess recurrence and long-term outcomes.

## Data Availability

Data supporting this study are available from the corresponding author upon reasonable request.
